# Integrative bioinformatics and machine learning approaches reveal oxidative stress and glucose metabolism related genes as therapeutic targets and drug candidates in Alzheimer’s disease

**DOI:** 10.3389/fimmu.2025.1572468

**Published:** 2025-06-26

**Authors:** Fatima Noor, Sidra Aslam, Ignazio S. Piras, Cecilia Tremblay, Thomas G. Beach, Geidy E. Serrano

**Affiliations:** ^1^ Institute of Molecular Biology and Biotechnology, The University of Lahore, Lahore, Pakistan; ^2^ Department of Pathology, Banner Sun Health Research Institute, Sun City, AZ, United States; ^3^ Neurogenomics Division, Translational Genomics Research Institute, Phoenix, AZ, United States

**Keywords:** oxidative stress, Alzheimer’s disease, glucose metabolism, bioinformatics, machine learning

## Abstract

**Background:**

Alzheimer’s disease (AD), the most common form of dementia, has treatments that slow but do not stop cognitive decline. Additional treatments are based on its pathogenic mechanisms are needed. Evidence has long highlighted oxidative stress and impaired glucose metabolism as crucial factors in AD pathogenesis. Therefore, in this study we aimed to find key AD pathogenic pathways combining genes involved in oxidative stress and glucose metabolism as well as potential small-molecule therapeutic agents.

**Methods:**

Using autopsy brain RNA sequencing data (GSE125583) derived from the Arizona Study of Aging and Brain and Body Donation Program, AD-related genes were identified via differential gene expression, pathway and coexpression analysis. Oxidative stress and glucose metabolism genes were correlated to pinpoint module genes. GSE173955 was used an independent dataset was used for validation, conducting molecular docking, assessing hub genes for AD, and integrating machine learning approaches.

**Results:**

We identified 13,982 differentially expressed genes (DEGs) in AD patients. Through WGCNA coexpression analysis, 1,068 genes were linked to AD-specific modules. Pearson’s correlation analysis highlighted 99 genes involved in oxidative stress and glucose metabolism. Overlap analysis of DEGs, module genes, and these metabolic genes revealed 21 key overlapping targets. PPI network and receiving operating curve (ROC) curve analyses then identified AKT1 and PPARGC1A as diagnostic hub genes for AD. Machine learning-based virtual screening of small molecules identified various inhibitors and enhancers with drug-like potential targeting AKT1 (upregulated) and PPARGC1A (downregulated), respectively. Among others, the Random Forest model was the most reliable for predicting molecular activity. Molecular docking further validated the binding affinities of these small molecules (inhibitors/enhancers) to AKT1 and PPARGC1A.

**Conclusion:**

This study identified AKT1 and PPARGC1A as potential therapeutic targets in AD. We discovered drug candidates with strong binding affinities, offering new avenues for effective AD treatment strategies.

## Graphical Abstract

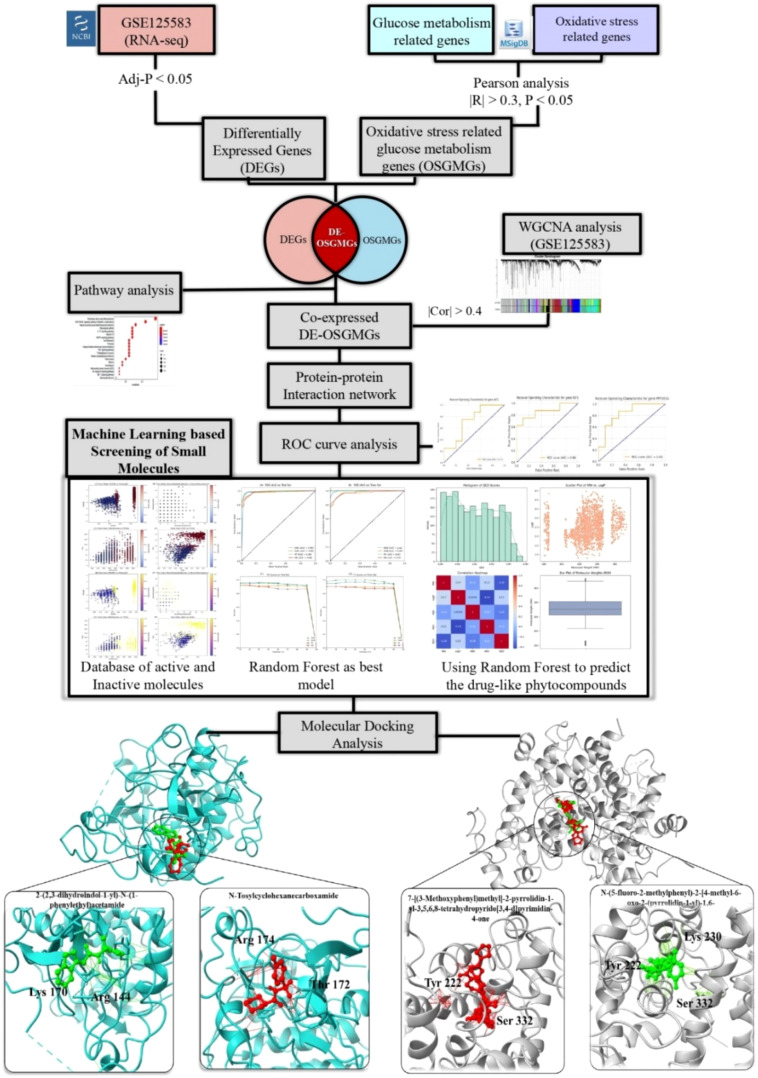

## 1 Introduction

Alzheimer’s disease (AD) is the most prevalent form of dementia globally and is a major cause of death among the elderly, significantly contributing to mortality and morbidity (1). It is reported that approximately more than 42.3 million people worldwide suffer from progressive cognitive impairment caused by AD (2). Further, the epidemiological analyses suggest that the number of people with AD will be more than double by 2060 (3). The gradual worsening of cognitive abilities in AD is not only a threat to the life quality of the patients, but also has social impacts and burdens on families and healthcare systems (4). The disease alters complex biochemical processes, particularly oxidative stress, and glucose metabolism, which both may contribute to the development of the disease and may be interconnected in such a manner as to promote neuronal damage (5, 6).

It is noteworthy that oxidative stress occurs when there is an excess of reactive oxygen species (ROS) as compared to the body’s capacity to detoxify these toxic substances or restore the damaged tissues (7). In AD, this may be further compounded by the dysregulation of genes involved in the oxidative stress response such as SOD, GPx, and catalases (8), leading to intraneuronal oxidative injury and apoptosis, possibly contributing to the cognitive impairment seen in AD (9, 10). At the same time, glucose metabolism, which is necessary for providing energy to the brain, may be significantly affected in AD (5, 11). The brain largely depends on glucose metabolism which is affected by defects in insulin signaling, glucose transport and glycolysis (12, 13). Metabolic dysfunction potentially results in decreased glucose availability, leading to the brain hypometabolism that is well documented in AD patients (14).

Beyond its effect on glucose metabolism, oxidative stress damages mitochondrial DNA and associated enzymes, ultimately decreasing ATP generation with resulting increased neuronal vulnerability (15). Additionally, impaired brain glucose metabolism in AD can lead to increased levels of oxidative stress due to enhanced ROS production (14). These two conditions potentially create a vicious cycle where each condition enhances the other. This has raised the need for more detailed research on the metabolic interactions within the AD brain, as this may translate to improved therapeutics.

This study attempts to unravel the complex nexus between oxidative stress and glucose metabolism in AD, shedding light on how these pivotal biochemical pathways influence the beginning and subsequent trajectory of the disease. By analyzing RNA-seq data, our study highlights critical metabolic disruptions that may underlie AD pathology. Our research uncovers essential co-expression networks and hub genes, spotlighting their significant roles in AD’s metabolic molecular landscape. This investigation not only deepens our understanding of the disease mechanisms but also opens new avenues for targeted therapeutic strategies, leveraging the potential of small molecules to modulate these key pathways.

## 2 Methodology

### 2.1 Data acquisition

**Figure 1:**
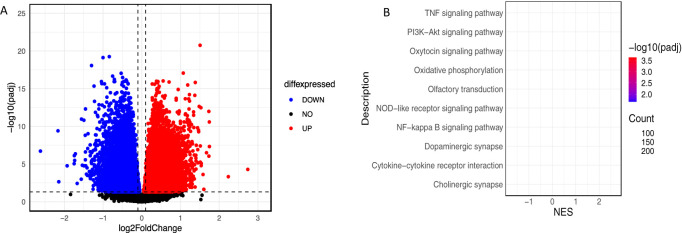
**(A)** Volcano plot to illustrate DEGs **(B)** KEGG enrichment analysis. The size of bubble indicates the number of genes involved in these pathways.

The RNA-seq data (GSE125583) used in this study was generated from the Arizona Study of Aging and Brain and Body Donation Program at Banner Sun Health Research Institute utilizing temporal lobe fusiform gyrus from 219 autopsy-confirmed cases and 71 age-similar controls from fusiform gyrus. These data available in GEO database of NCBI (http://www.ncbi.nih.gov/geo) were used as training set, while the GSE173955 dataset (16), which includes a total of 18 biological samples consisting of 8 cases from AD patients and 10 cases from non-Alzheimer’s controls was used for independent validation (test dataset). Further details of the methods used to generate and analyze this data has been previously published (17). Gene expression profiles were set based on two parameters 1) Tissues samples collected from diseased fusiform gyrus tissue and normal fusiform gyrus tissue, (2) number of samples were obtained for each dataset were more than 3. Although the validation dataset (GSE173955) includes a relatively small sample size (n=18), its inclusion aligns with precedents in transcriptomic research, where datasets with >3 biological replicates per group have been effectively used for validation purposes (18). Given the biological relevance and consistent tissue context, this dataset provided a valuable reference point to support the reproducibility of our findings across independent cohorts. MsigDB (https://www.gsea-msigdb.org/gsea/msigdb/) and Genecards (https://www.genecards.org/) databases retrieved the oxidative stress and glucose metabolism gene list using keywords “oxidative stress” “glucose metabolism”, and “Glucometabolic”. The flowchart of study is shown in **Figure 1**.

### 2.2 Analysis of gene expression variability and pathway enrichment in AD

Differentially expressed genes (DEGs) between AD and controls were identified using the DESEQ2 package in R with the cutoff point adjusted p-value < 0.05. To illustrate the DEGs, volcano plots were created using the R package ggplot2. KEGG pathway analysis was conducted on DEGs to investigate their functional enrichment using the *clusterProfiler R-*package. False discovery rate < 0.05 was selected as cutoff criterion to represent significant enrichment.

### 2.3 Coexpression analysis using WGCNA and identification of key modules

WGCNA analysis (19) was conducted on dataset GSE125583 to find the AD associated significant coexpression network and key modules. DESEQ2 was used to normalize the counts table. After that, we computed the median absolute deviation (MAD) to calculate the variability of genes and retained only the 50% of high variable genes based on MAD score, eliminating the low variable genes causing noise in the coexpression analysis. Soft-thresholding value (β) was computed using pickSoftThreshold function and the lowest value was selected when scale free topology index curve flattens out upon reaching r^2^ = 0.90 (20). We constructed a signed coexpression network and recognized the resulting clusters utilizing the function *blockwiseModules* with the following parameters: *TOMtype*: “signed”, *deepSplit* = 2, *minimum module size* = 30, *mergeCutHeight* = 0.30, and *pamRespectsDendro* = “TRUE”. We calculated the eigengene values for each individual and module using singular value decomposition (SVD) (21). We compared the eigengenes by module between AD and control using a linear model implemented in *limma*. Covariates were not included in the model since we used the adjusted expression matrix. To account for multiple testing, we adjusted the p-values by accounting for the number of modules using the FDR approach. The analysis was conducted using the WGCNA R-package. Modules that correlated the most with the clinical trait which includes disease phenotype (AD vs control) were labeled as AD-related modules. We only select those modules whose correlation with AD trait was greater than 0.4 and then used these to explore the correlation between module membership (MM) and gene significance (GS) to find the key AD-related modules.

### 2.4 Selection of genes related to glucose metabolism and oxidative stress

To find significant oxidative stress-related glucometabolic genes (OSGMGs), the expression levels of glucose metabolism related genes were compared with oxidative stress related genes to calculate Pearson correlation coefficient using the cor.test() function in R with the threshold set at p<0.05 and |r|>0.4.

### 2.5 Intersection genes and Venn analysis and hub gene prediction

We utilized a Venn diagram drawing tool (http://bioinformatics.psb.ugent.be/webtools/Venn/) to create Venn diagrams, representing the overlap between DEGs, WGCNA-derived key module genes and OSGMGs genes. Intersection genes were included in subsequent analyses. The STRING database (http://www.string‐db.org/), which searches for known and predicted interactions between proteins, was used to construct the protein-protein interaction network between 21 genes. The resulting network was then visualized using Cytoscape 3.9.1.

### 2.6 ROC analysis

ROC curve analysis was performed to check the prognostic efficiency of five hub genes. Only those genes with AUC greater than 0.7 were retained for further study (22).

### 2.7 Machine learning-based virtual screening of small molecules

#### 2.7.1 Data preparation and preprocessing

The study commenced with the uploading of active and inactive molecules of target proteins. These molecular structures were described using SMILES (Simplified Molecular Input Line Entry System) notation. The RDKit library (23), a cheminformatics toolkit, was employed to convert these SMILES strings into RDKit Mol objects, as required for molecular descriptor calculations. Potential decoys were removed from the library of small molecules to get a balanced data set of equal number of actives and inactives depending on molecular weight. The final dataset was then shuffled to avoid any order influence during the training of the model.

#### 2.7.2 Feature engineering and descriptor calculation

The approach involved calculating various molecular descriptors to characterize and differentiate the active from inactive molecules. RDKit’s Descriptors and GraphDescriptors modules were employed to compute a wide array of features, including molecular weight, logP, partial charges, EState indices, and Morgan fingerprints, among others. These descriptors capture different aspects of chemical structure and properties, essential for effective modeling of biological activity. To address missing values, a mean imputation strategy was implemented. Subsequently, Principal Component Analysis (PCA) was performed to reduce dimensionality while retaining critical variance in the data, facilitating more efficient and insightful modeling (24).

#### 2.7.3 Model development and selection

The preprocessed data (containing both active and inactive molecules of target proteins) was partitioned into training and testing sets, ensuring a 70:30 split to ensure that there is a strong evaluation criterion in place. Several machine learning algorithms were then used for training, namely k-Nearest Neighbors (kNN) (25), Support Vector Machines (SVM) (26), Random Forest (RF) (27), Naive Bayes (NB) (28), and Gradient Boosting (GB) (29) Classifiers. Parameter tuning of each model was done using GridSearchCV for the best hyperparameters setting. To evaluate model performance on unseen data, Stratified K-Fold cross-validation was used. The choice of the model was made depending on the accuracy, sensitivity, specificity, MCC, and AUC values (30). The final model was then used to screen a library of small molecules (including both inhibitors and enhancers).

#### 2.7.4 Virtual screening and drug-likeness prediction

Choosing one model, a list of small molecules was filtered (including both inhibitors and enhancers) to determine which molecules had the highest probability of inhibiting/enhancing the target protein. The list of hits was then narrowed down by using the drug-likeness criteria defined by Lipinski’s Rule of Five. This rule measures the drug likeness for a molecule in terms of molecular weight, hydrogen bond donors and acceptors, and lipophilicity (logP). Those molecules that fulfilled these criteria were regarded as potential drug-like scaffolds and were transferred to the next stage of testing. In adopting this strategy, the study sought to screen for new drugs with possible therapeutic benefits rapidly and cost-effectively.

#### 2.7.5 Molecular docking analysis

In the present work, molecular docking was used to examine the binding mode of the target proteins with the small molecules to assist in the identification of appropriate drug combinations for increasing disease treatment effectiveness. The first 3D structures of target proteins included in this study were obtained from the Research Collaboratory for Structural Bioinformatics (RCSB) Protein Data Bank, a public database containing structural information of biological macromolecules (http://www.rcsb.org/pdb/) (31). The docking studies were conducted using Autodock vina 1.1.2 within PyRx 0.8 (32), employing the predicted X-ray crystal structure of key proteins and the active components. The SMILES formats of the compounds were sourced from PubChem and converted using OpenBabel integrated into the PyRx platform. A total of 2000 steps was set for energy minimization, ceasing when the energy differential was less than 0.01 kcal/mol, to achieve a stable conformation. Subsequently, the compounds/ligands were converted into the.pdbqt format for docking analyses. The active site residues of the target proteins were identified with the CASTp tool (33). PyRx 0.8 facilitated the computation of binding affinities between the small molecules and target proteins. The most favorable docked complexes were selected based on their Root Mean Square Deviation (RMSD) and binding energies, with values of less than -5.00 kcal/mol indicating strong binding, and less than -7.00 kcal/mol signifying very strong binding (34). The RMSD calculation serves as a measure of how much the docked conformation deviates from this reference structure, with lower RMSD values indicating a closer match to the expected or known binding conformation. Complexes with the lowest RMSD values were considered the most favorable, as they suggest the least deviation from the reference, implying a high degree of accuracy in reproducing known or theoretically optimal binding poses. Finally, the visual representation of the docked complexes was performed using Discovery Studio (35), PyMOL (36), and ChimeraX (37) programs.

## 3 Results

### 3.1 Identification and functional enrichment analysis of DEGs in AD

A differential expression analysis of the dataset GSE125583 identified 13,982 genes that were differentially expressed in AD, including 7,131 upregulated and 6,851 downregulated genes (**Figure 1A**) (adj-p < 0.05). Further, we performed Gene Set Enrichment Analysis on the same dataset. Results showed that there were significant functional class enrichments among the DEGs, which were mainly enriched for pathways related to oxidative phosphorylation, synaptic pathways, and signaling pathways in AD (**Figure 1B**).

### 3.2 Weighted expression network construction to identify key modules

**Figure 2:**
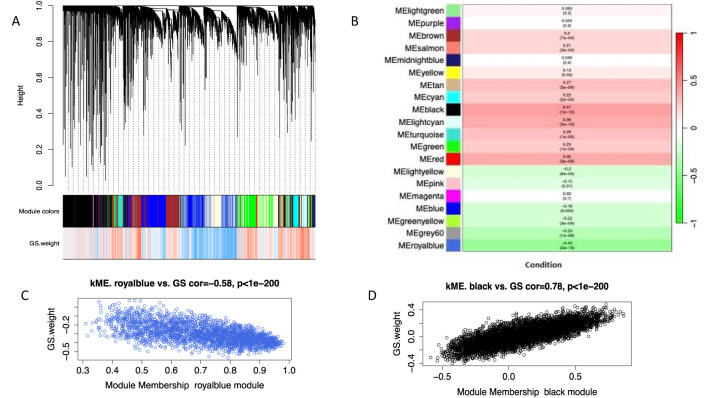
WGCNA analysis of GSE125583. **(A)** Cluster dendrogram of co-expression weighted Correlation network analysis (WGCNA) indicated through different colors **(B)** Module-trait relationships; correlation between module eigengenes (ME) and disease phenotype **(C)** Key module in royalblue represents highly negative correlation with disease phenotype **(D)** Key module in Black showed highly positively correlation with disease phenotype.

A total of 20,000 genes were retained based on MAD score derived from the 289 samples of the GSE125583 dataset. These genes were used to construct a co-expression network. We obtained a total of 20 coexpression modules, with the number of genes in each module ranging from 214 (lightgreen module) to 5624 (black module) (**Figure 2A**). After eigengene extraction and differential analysis, 16 modules were associated with AD (**Figure 2B**). Only two modules (royalBlue, black) were retained as key modules (**Figures 2C, D**) (Pearson’s r > 0.4). A total of 1068 genes were found in the two AD-associated modules, which were used for further analysis.

### 3.3 Selection of genes related to glucose metabolism and oxidative stress

Based on the MsigDB and Genecards databases, we obtained 1,399 oxidative stress-related genes and 1,005 glucose metabolism-related genes. Pearson’s correlation between glucose metabolism and oxidative stress relative genes, with the threshold set at p<0.05 and |r|>0.4, identified 99 oxidative stress and glucose metabolism-related genes (OSGMGs).

### 3.4 Intersection between DEGs, WGCNA, OSGMGs, and PPI network construction

**Figure 3:**
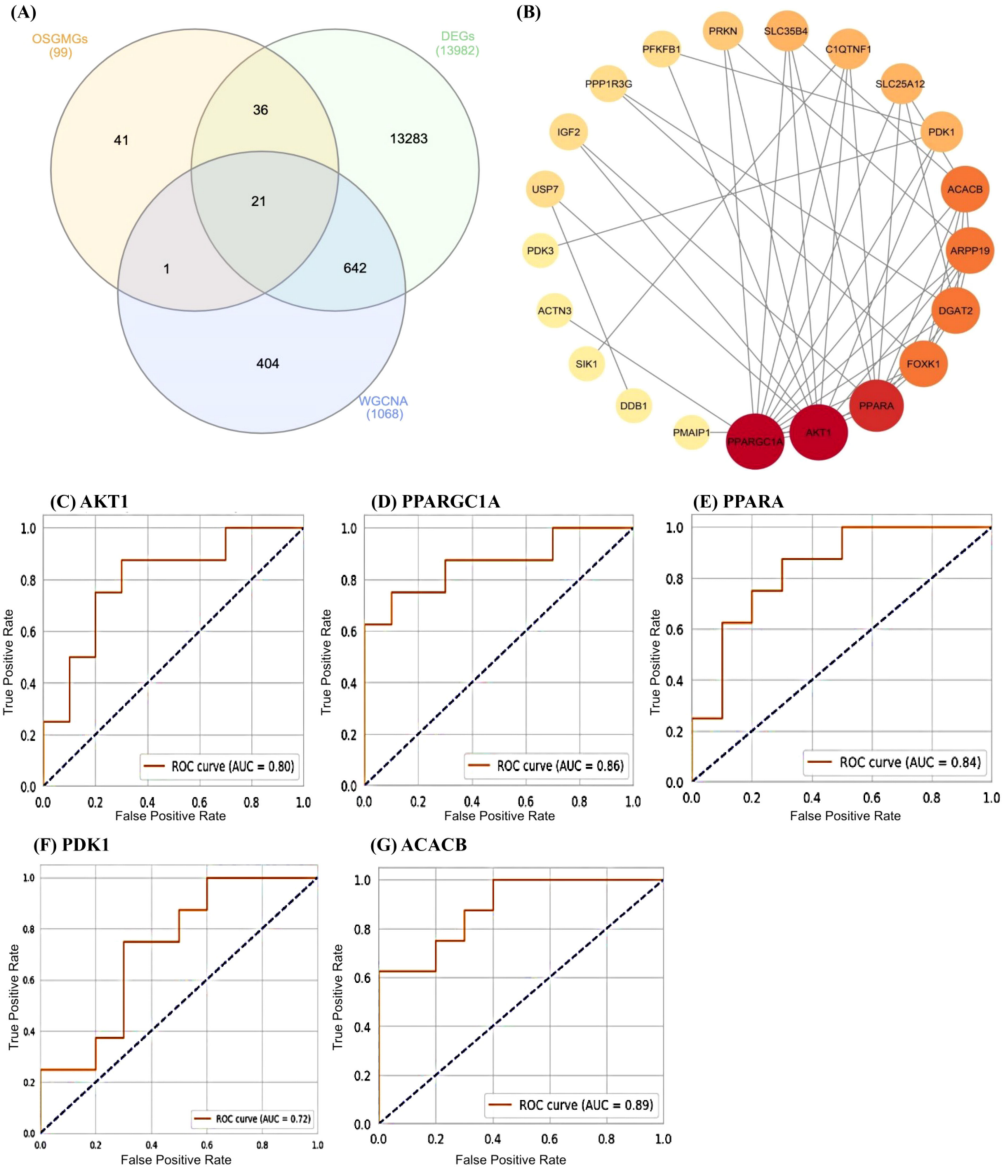
**(A)** A Venn diagram showing the overlap between three different groups: OSGMGs (99), DEGs (13982), and WGCNA (1068). **(B)** Protein-Protein Interaction (PPI) network for 21 common genes identified in the Venn diagram. The nodes (circles) represent genes, and the edges (lines) indicate interactions between them. The size of the node and its color intensity indicate the level of connectivity, with larger, darker nodes having more connections. **(C-G)** These are Receiver Operating Characteristic (ROC) curves for five different genes: AKT1, PPARGC1A, PPARA, PDK1, and ACACB.

By taking the intersection of the DEGs, WGCNA-based key module genes, and OSGMGs genes, 21 overlapping genes were identified (**Figure 3A**). STRING database was used to construct the PPI network to assess the interactions between proteins corresponding to the DEGs at a combined score > 0.7. The network was visualized using Cytoscape software, consisting of 21 nodes and 45 edges (**Figure 3B**).

### 3.5 Identification and validation of hub genes through ROC analysis

The PPI network including 21 genes was analyzed using the CytoHubba plugin in Cytoscape, with the goal to identify the most influential hub genes. From this network, the top five hub genes (AKT1, PPARGC1A, PPARA, PDK1, and ACACB) were selected for further validation. Subsequent ROC analysis was performed to evaluate their prognostic utility (**Figure 3**). Among these, two genes, AKT1 and PPARGC1A, demonstrated high predictive accuracy with AUC values exceeding 0.70, indicating their potential involvement in AD pathogenesis or progression.

### 3.6 Machine learning-based virtual screening of small molecules

#### 3.6.1 Data collection and processing

**Figure 4:**
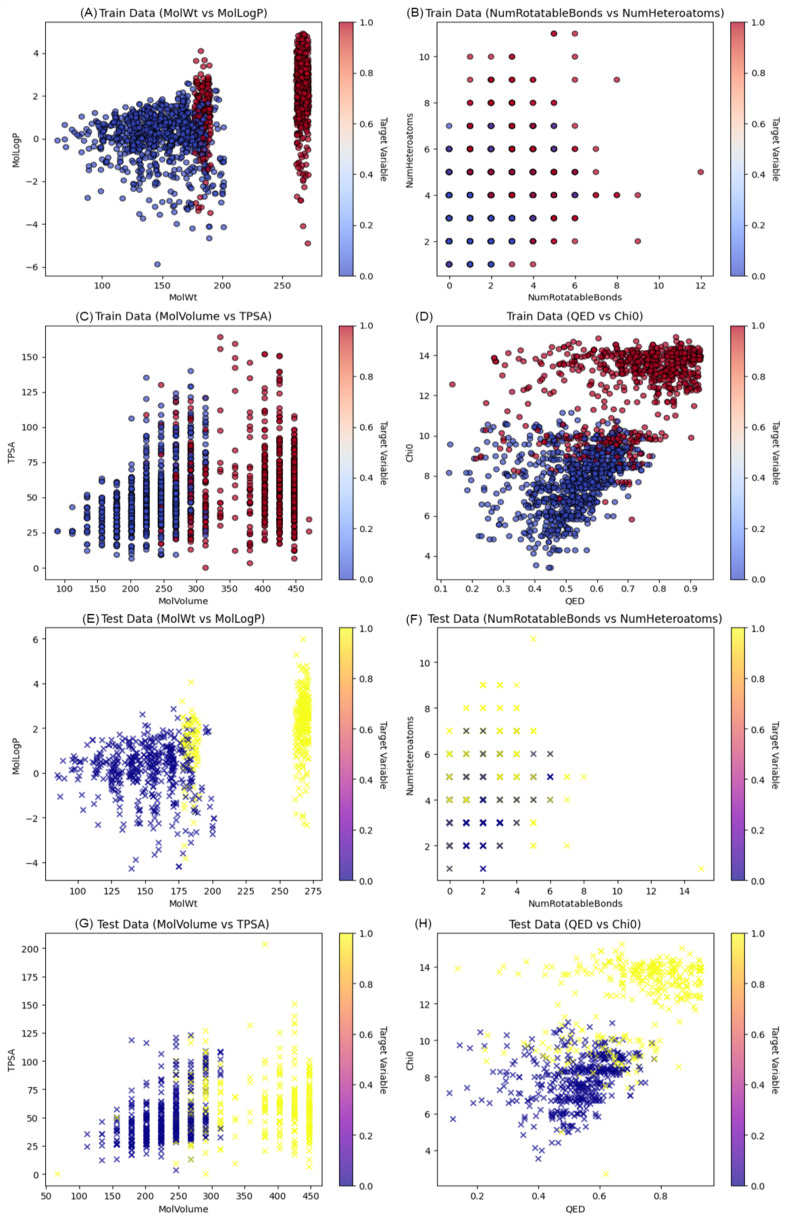
Visualization of molecular descriptors and target variable distribution across training and test datasets. **(A)** Molecular Weight (MolWt) vs. MolLogP, illustrating the distribution of lipophilicity across a range of molecular sizes. **(B)** Number of Rotatable Bonds vs. Number of Heteroatoms, capturing molecular flexibility and polarity characteristics. **(C)** Molecular Volume vs. TPSA (Topological Polar Surface Area), reflecting size and surface polarity. **(D)** QED (Quantitative Estimate of Drug-likeness) vs. Chi0 (connectivity index), highlighting molecular quality and complexity. **(E)** MolWt vs. MolLogP **(F)** NumRotatableBonds vs. NumHeteroatoms **(G)** MolVolume vs. TPSA **(H)** QED vs. Chi0.

Following the identification and validation of hub genes through ROC analysis, we conducted machine learning-based virtual screening of small molecules. Data collection and processing involved compiling a list of active and inactive molecules associated with the AKT1 and PPARGC1A proteins. After obtaining the molecular structures, a rich set of features was defined to describe the chemical properties of each molecule. This allowed the creation of a dataset suitable for the use of machine learning algorithms in the context of molecular activity prediction. The active and inactive molecules dataset was then split into a training set and test set to enable the building and assessment of the predictive models. The former was used to optimize the parameters of the models and the latter was used to evaluate their accuracy. The scatter plots shown in **Figure 4** illustrates the correlation between different chemical features in the training set. These maps are useful for identifying the patterns of distribution of molecular descriptors and their possible relationships. The observed pattern and distribution in the training data can be explained by some chemical and physical properties that could be used to increase the prediction accuracy of the model. The results of the study indicate that some of the descriptors like molecular weight and LogP have the potential to influence the molecular activity predictions. While other features can be less clear as to how they contribute to the model’s ability to make accurate predictions and may need further explanation or may have to be combined with other descriptive features.

#### 3.6.2 Principle component analysis

**Figure 5:**
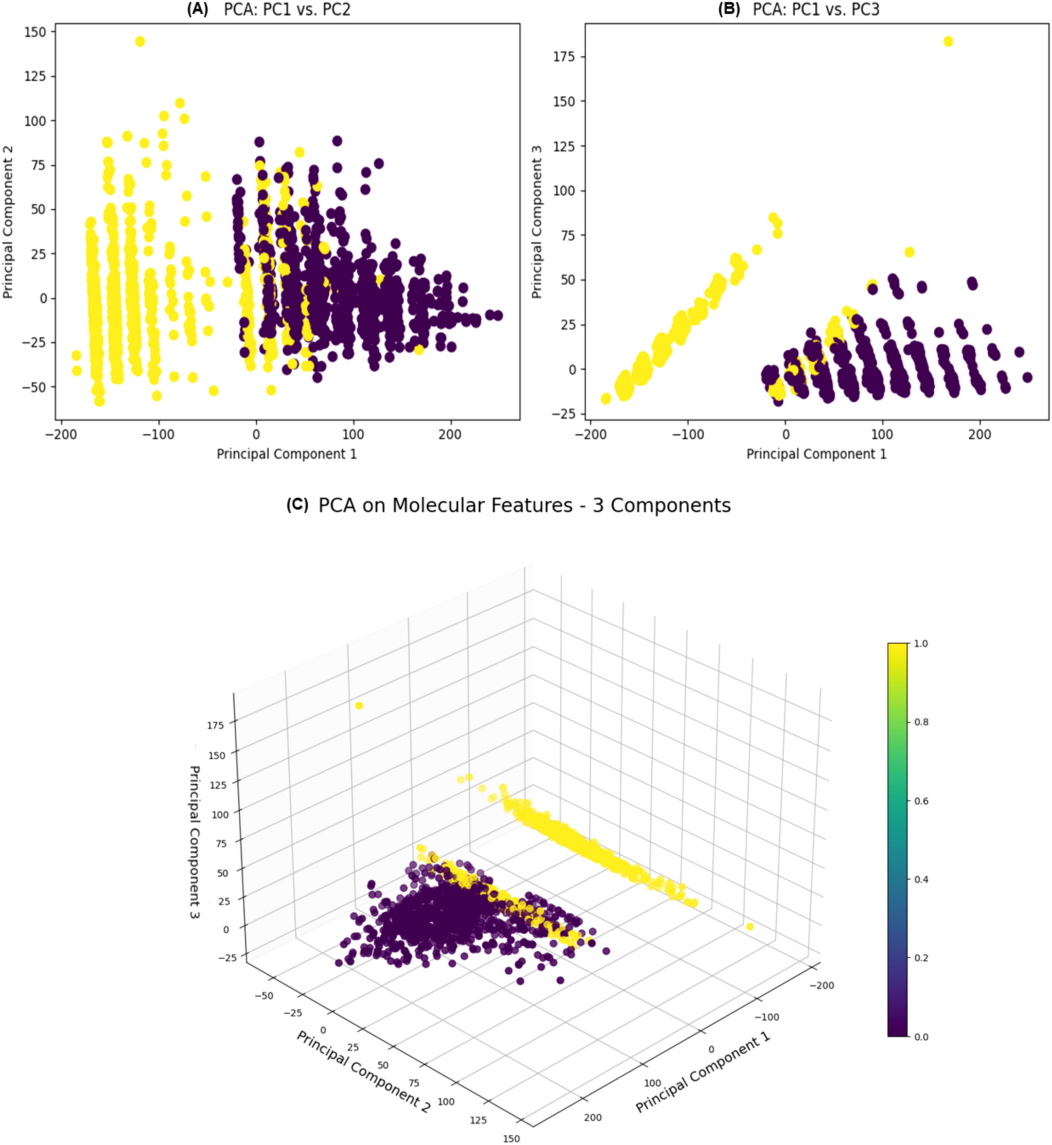
Two 2D scatter plots illustrating the relationships between **(A)** Principal Component 1 (PC1) and **(B)** Principal Component 2 (PC2) (left) and between Principal Component 1 (PC1) and Principal Component 3 (PC3) (right) are displayed. **(C)** A 3D scatter plot illustrates the relationships between the PC1, PC2, and PC3 obtained through PCA on molecular features. Each data point is color-coded according to the ‘Label’ column and uses the ‘viridis’ color map. The size of data points is standardized to 50.

The Principal Component Analysis (PCA) applied to the molecular features resulted in a distinct distribution of variance across the components (**Figure 5**). The first principal component (PC1) explained an overwhelming majority of the variance, at 98.9%, with an eigenvalue of 2301, signifying its dominance in capturing the data’s variability. The second principal component (PC2), although explaining a much smaller fraction of the variance at 0.0358% and having an eigenvalue of 0.4925, still contributes additional, albeit minor, details about the data’s structure. Given these values, PC1 is the primary axis of variation, providing a strong indication that it encapsulates the essential patterns within the molecular features. PC2 and any subsequent components, such as PC3, explain progressively less variance, indicating that they capture more subtle and complex relationships in the dataset. The transformed data, with its reduced dimensionality, was further utilized for in-depth analysis. The concentration of variance in PC1 facilitated a more streamlined and focused examination of the molecular properties that are most influential in determining compound activity. This transformation is instrumental for enhancing the efficiency and accuracy of the predictive modeling process, enabling the identification of compounds with desired biological activities. The PCA results thereby provide a robust foundation for the next stages of analysis, including the training of machine learning models to classify the compounds effectively.

#### 3.6.3 Model evaluation

**Figure 6:**
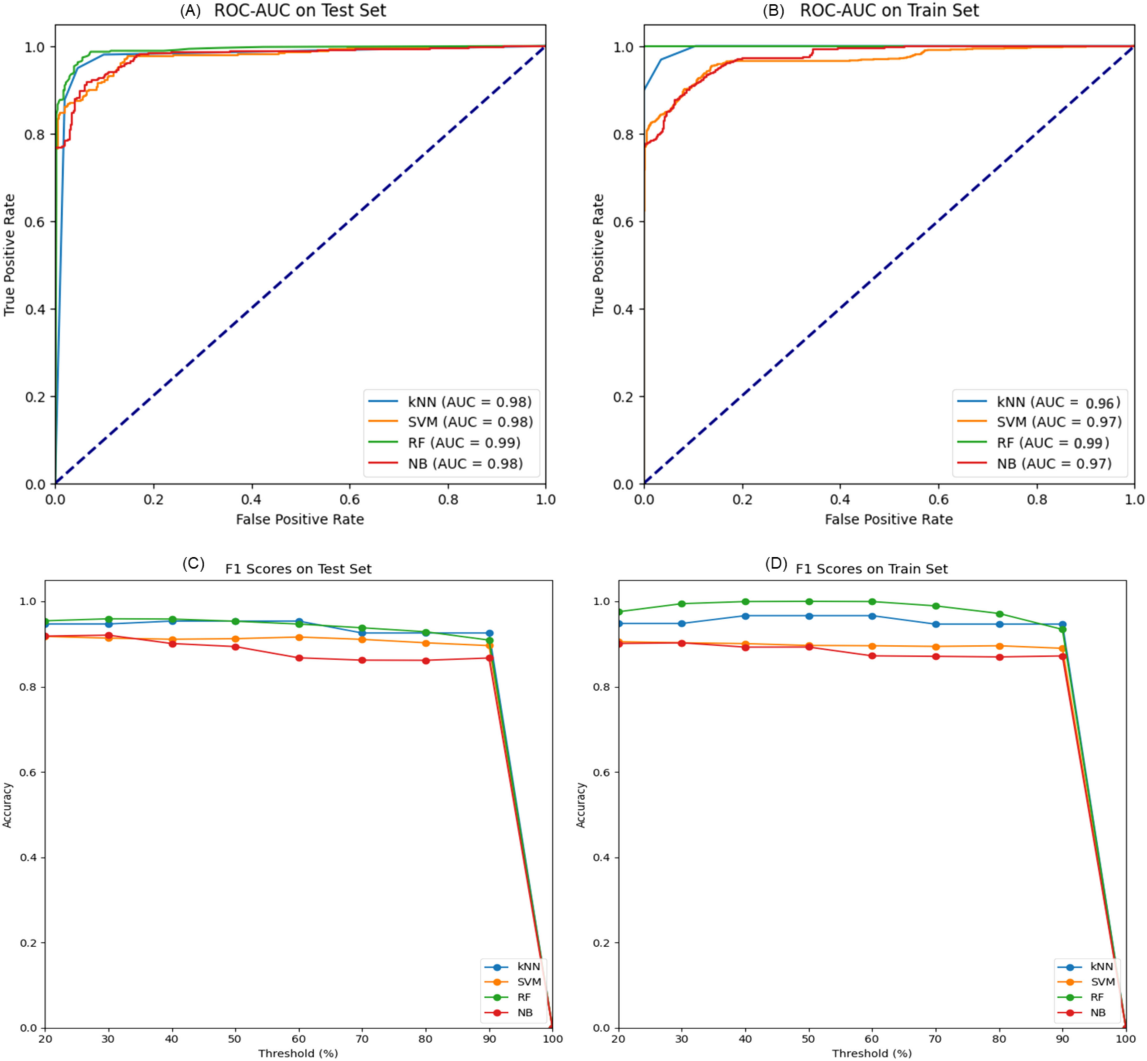
**(A)** ROC-AUC curves on the test set for k- Nearest Neighbors (kNN), Support Vector Machine (SVM), Random Forest (RF), and Naive Bayes (NB) models demonstrate high predictive accuracy where AUC values are presented. **(B)** The ROC-AUC curves on the training set indicate how well the models capture the underlying patterns. **(C, D)** show the F1 scores against the decision threshold for the test and training data, respectively, making it easier to understand the precision-recall curves and the ability of models to generalize different thresholds.

The performance of four machine learning models—k-Nearest Neighbors (kNN), Support Vector Machine (SVM), Random Forest (RF), and Naive Bayes (NB) was meticulously assessed using a dataset partitioned into training and test subsets. ROC-AUC and F1 scores were used as the key performance indicators for the models’ discriminative capacity and, correspondingly, the balance between precision and recall. On the test dataset, the Random Forest model stood out as the best-performing classifier with an AUC of 0. 990 indicating a better prediction ability of the model. This performance was closely followed by kNN and NB models, both having impressive AUC value of 0. 98 on the test set. 98 on the test set. Even though the SVM model has AUC of 0. 975, it still offered a good amount of accuracy (**Figure 6**).

The evaluation of F1 score for all the models at different threshold indicated that all the models performed well for both for test and training set and the reliability of their predictions. In the context of further metrics like accuracy, sensitivity, specificity, and MCC, additional peculiarities of every model became clear. The kNN model provided a good balance with a high accuracy of 95.17% and sensitivity 94.95%. While SVM model demonstrated a satisfactory specificity of 94.07%. In addition to the improved AUC, the RF model demonstrated high accuracy and specificity, with MCC being 0. 904 which proved its good predictive ability. However, the Naive Bayes classifier, which has the least mean accuracy of the four (89. 65%) and still retain a high specificity of 96 (**Table 1**). The ARI was moderately high at 0. In conclusion, the Random Forest model was outstanding in most of the criteria hence, it was the most accurate model for this data set. However, the high AUCs for all models imply that each one of them could be used for different forms of prediction roles depending on the level of sensitivity and specificity necessary.

**Table 1:** Comparative performance metrics of machine learning models for classification.

Model	Accuracy	Sensitivity	Specificity	MCC	AUC
kNN	0.951724	0.949451	0.954217	0.903323	0.976097
SVM	0.917241	0.859341	0.980723	0.842064	0.975628
RF	0.951724	0.942857	0.961446	0.903585	0.990369
NB	0.896552	0.832967	0.966265	0.802135	0.975718

#### 3.6.4 Quantitative evaluation of a small molecules for drug likeness and molecular property analysis

**Figure 7:**
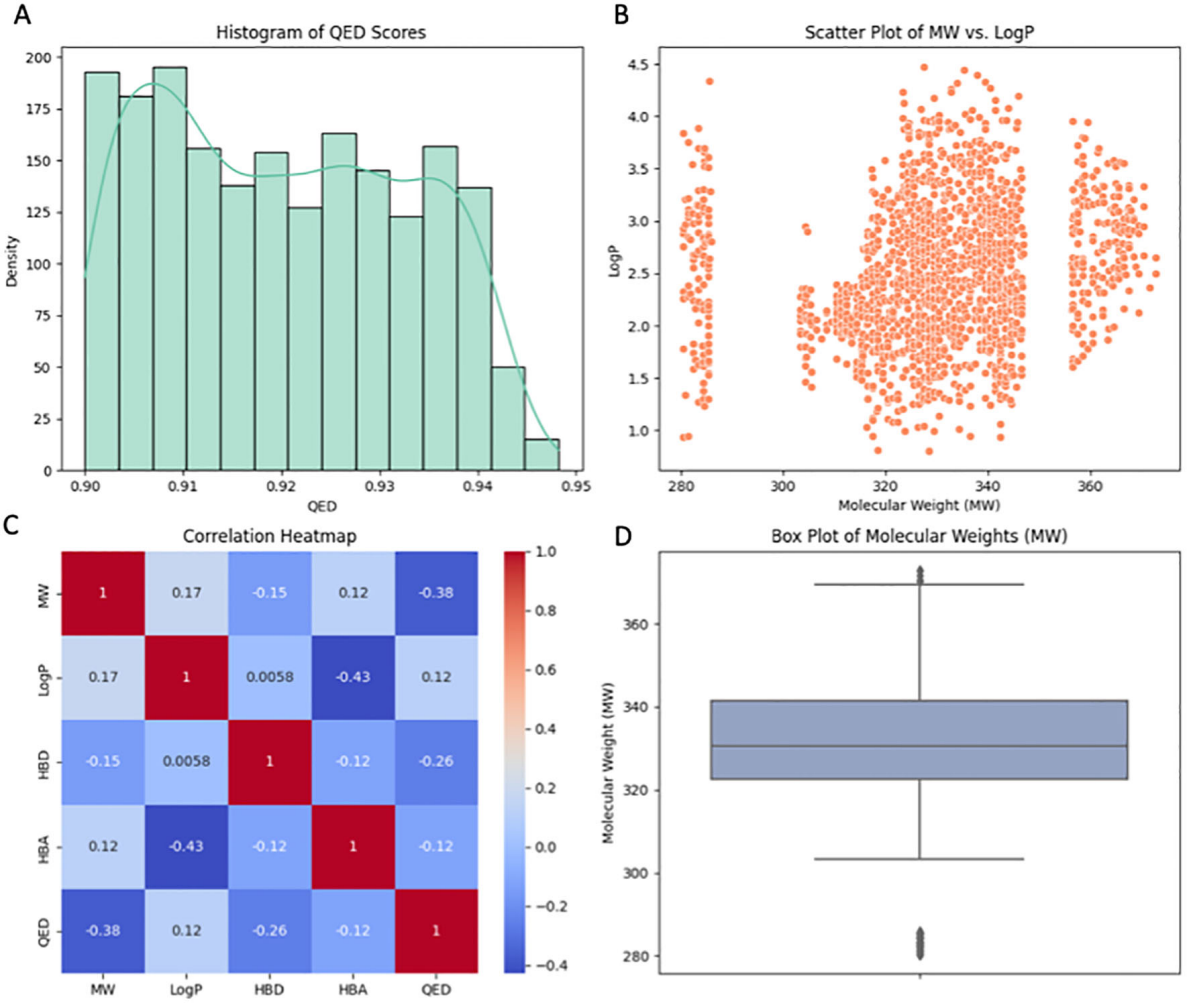
Visualization of key properties and relationships among filtered molecules. **(A)** Histogram displaying the distribution of Quantitative Estimation of Drug-likeness (QED) scores, with a density curve indicating the probability density. **(B)** Scatter plot illustrating the correlation between Molecular Weight (MW) and the partition coefficient (LogP), revealing patterns in MW vs. LogP. **(C)** Correlation heatmap revealing the relationships between molecular properties, with darker shades indicating stronger correlations. **(D)** Box plot presenting the distribution of Molecular Weights (MW) for the small molecules, highlighting central tendencies and potential outliers. Enhanced colors and styles enhance the visual appeal of the plots.

The Random Forest (RF) model was used to screen library of molecules (inhibitors/enhancers) to identify molecules that would yield the desired selection criteria. These criteria were based on the RF model’s predictive outcomes and the compounds’ compliance with the five rules referred to as Lipinski’s rule of five, which include the molecular weight (MW), the number of hydrogen bond donors (HBD), the number of hydrogen bond acceptors (HBA), and the octanol-water partition coefficient (LogP). To compare the distribution of QED scores, which is the measure of drug-likeness of molecules, histogram was plotted. Most of the molecules in the library were confirmed to bear high QED scores proving their drug like properties. A library of molecules with concentrations within the range of 0.90 to 0.95 was observed, indicating a promising segment of the library that can possibly be nurtured further. A scatter plot of MW against LogP revealed a wide distribution of molecules across different ranges of lipophilicity and molecular weight (**Figure 7**). This plot demonstrates the diversity of the library in terms of these two important descriptors. Despite the spread, no clear trend was observed, suggesting the presence of both lipophilic and hydrophilic molecules across various molecular weights. The box plot of molecular weights highlighted the central tendency and the dispersion of the MW data within the library. The plot showed a relatively tight interquartile range, indicating that the majority of the molecules had molecular weights within a narrow window, which is consistent with typical drug-like molecules. The correlation heatmap provided insights into the relationships between different molecular descriptors. Notably, QED scores were negatively correlated with molecular weight, suggesting that as the molecular weight increases, the drug-likeness as per the QED scores tends to decrease. Other descriptors such as HBD and HBA showed varying degrees of correlation with LogP and QED, offering a nuanced view of how these properties interplay in the context of drug discovery. In conclusion, the comprehensive analysis utilizing the RF model and adherence to Lipinski’s rule of five successfully narrowed down a vast library to molecules with QED scores above 0.90. These selected molecules, representing a drug-like profile, are earmarked for further in-depth docking analysis to assess their potential as therapeutic agents. The use of such stringent selection criteria ensures that the candidates progressing to the docking stage have a higher likelihood of exhibiting favorable pharmacokinetic and pharmacodynamic properties.

#### 3.6.5 Validation using molecular docking

**Figure 8:**
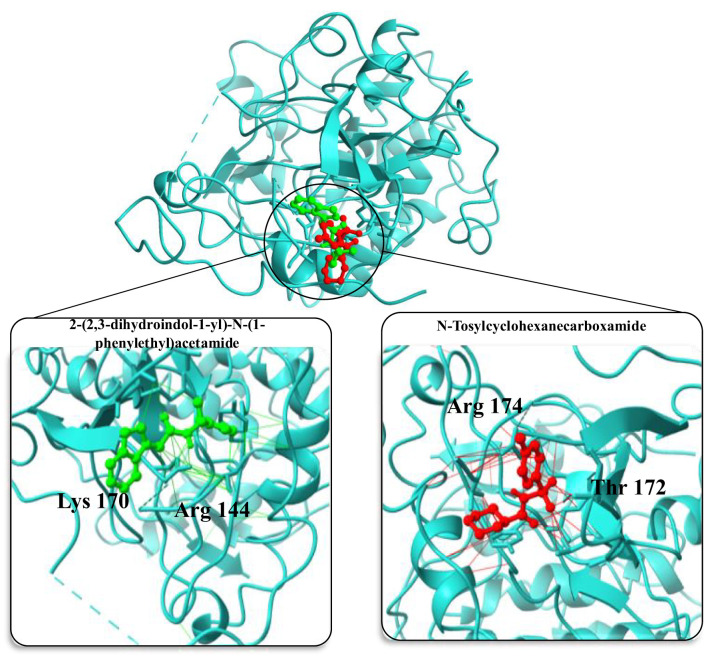
Structural representation of AKT1 protein with inhibitor binding sites. The main image showcases the overall tertiary structure of the protein with two inhibitor molecules bound at distinct sites. Insets provide detailed views of the binding interactions: the left inset highlights 2-(2,3-dihydroindol-1-yl)-N-(1-phenylethyl)acetamide (in green) interacting with amino acids Arg 144 and Asp 170, while the right inset shows N-Tosylcyclohexanecarboxamide (in red) in proximity to Arg 144 and Thr 172. These interactions are crucial for the inhibitory mechanism and provide insights into the molecular architecture of the binding pockets within AKT1.

In the molecular docking study conducted to evaluate potential inhibitors of the upregulated AKT1 protein, a series of small molecules were analyzed for their binding affinities and conformational fits within the protein’s active site (**Figure 8**). The molecule 2-(2,3-dihydroindol-1-yl)-N-(1-phenylethyl) acetamide exhibited a substantial binding affinity of -7.4 kcal/mol and a conformational RMSD of 2.1 Å, indicating a promising interaction with AKT1. Another notable molecule, N-Tosylcyclohexanecarboxamide, demonstrated an even higher binding affinity of -8.3 kcal/mol coupled with a lower RMSD of 1.4 Å, positioning it as a strong candidate for further investigation as an AKT1 inhibitor. Further, 1-methyl-N-[[2-(2-methyloxan-2-yl) pyrimidin-5-yl]methyl]pyrazole-3-carboxamide also showed favorable binding characteristics with a -7.2 kcal/mol affinity and a 2.1 Å RMSD (**Table 2**). However, N-cyclopentyl-5-(2-furyl)-2-methyl-2H-1,2,6-thiadiazine-3-carboxamide 1,1-dioxide presented an atypical positive binding affinity of 7.3 kcal/mol, suggesting it may not function effectively as an inhibitor under the tested conditions. Lastly, N-[(2-methoxyphenyl) methyl]-1-pyrazin-2-ylpiperidine-3-carboxamide displayed a lower binding affinity of -6.3 kcal/mol and an RMSD of 2.8 Å, which, despite being the least promising among the candidates in terms of binding affinity, could still be considered for optimization due to its distinct chemical structure. These findings highlight the potential of N-Tosylcyclohexanecarboxamide as a lead molecule, given its strong binding affinity and structural stability within the AKT1 binding site, while also acknowledging the need for further experimental studies to validate the computational predictions and to optimize the binding efficiencies of the other small molecules.

**Table 2:** Molecular docking results for AKT1 with screened molecules.

Inhibitors	PubChem IDs	Binding Affinity (kcal/mol)	RMSD
2-(2,3-dihydroindol-1-yl)-N-(1-phenylethyl)acetamide	5203469	-7.4	2.1
N-Tosylcyclohexanecarboxamide	4879102	-8.3	1.4
1-methyl-N-[[2-(2-methyloxan-2-yl)pyrimidin-5-yl]methyl]pyrazole-3-carboxamide	110117862	-7.2	2.1
N-cyclopentyl-5-(2-furyl)-2-methyl-2H-1,2,6-thiadiazine-3-carboxamide 1,1-dioxide	22515296	7.3	2.1
N-[(2-methoxyphenyl)methyl]-1-pyrazin-2-ylpiperidine-3-carboxamide	23607864	-6.3	2.8

**Figure 9:**
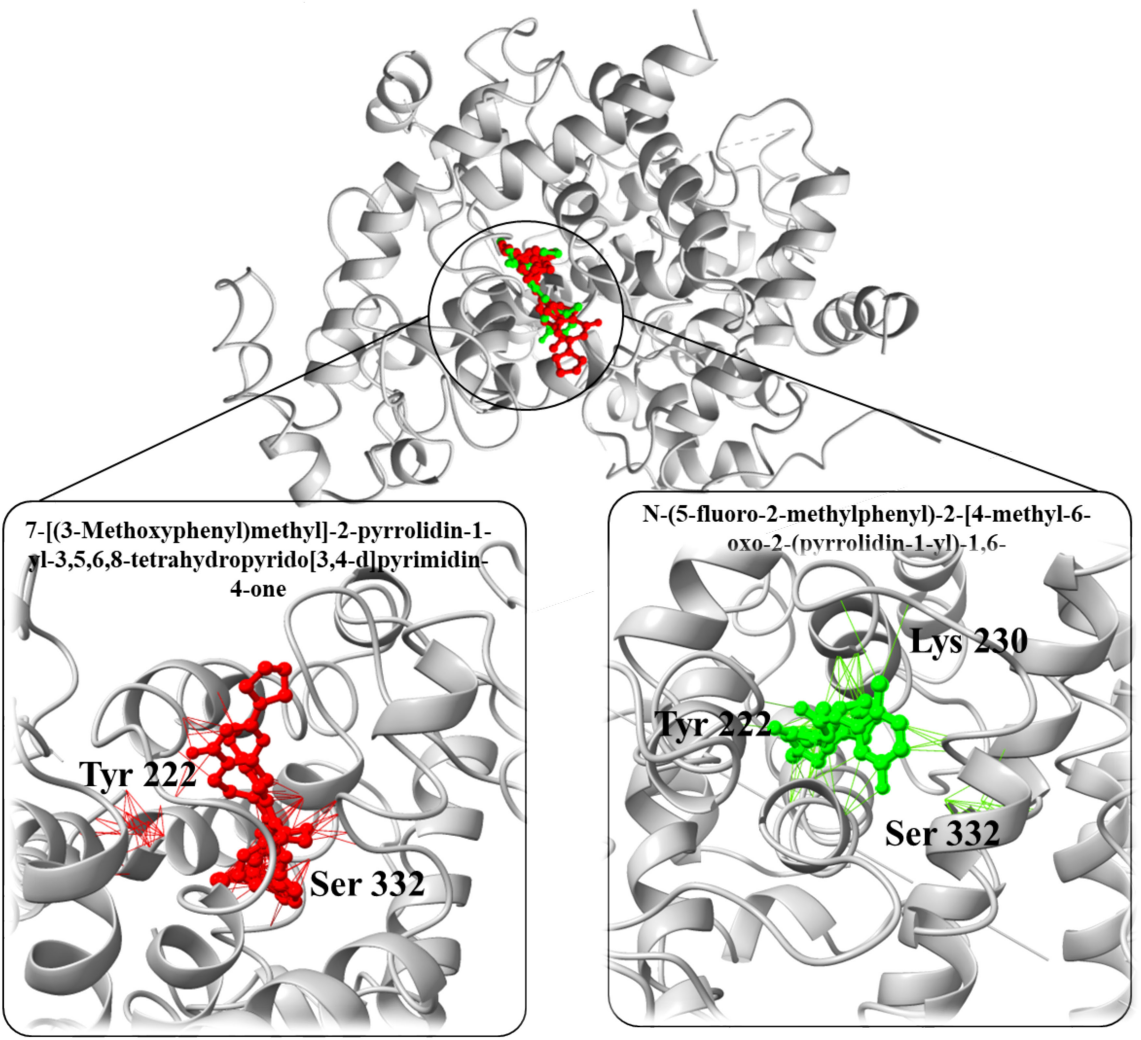
Three-dimensional structure of the PPARGC1A protein with bound ligands. The central protein structure is shown in a ribbon diagram with two ligands bound in distinct active sites. The left inset details the interaction of the ligand 7-[(3-Methoxyphenyl) methyl]-2-pyrrolidin-1-yl-3,5,6,8-tetrahydropyrido[3,4-d] pyrimidin-4-one (in red) with key amino acids Tyr 222 and Ser 332. The right inset shows the ligand N-(5-fluoro-2-methylphenyl)-2-(4-methyl-6-oxo-2-(pyrrolidin-1-yl)-6H-pyridazin-1-yl) acetamide (in green) in proximity to Tyr 222, Lys 230, and Ser 332, illustrating the ligands’ positions relative to important residues within the binding domain of PPARGC1A.

In a parallel molecular docking study targeting the downregulated PPARGC1A protein, various small molecules (enhancers) were evaluated to ascertain their potential as enhancer (**Figure 9**). The enhancer 1-(3,4-dihydro-2H-pyrrol-5-yl)-3-(2-methoxyphenyl)-1-(4-methoxyphenyl)urea stood out with a binding affinity of -9.1 kcal/mol and an RMSD of 1.1 Å, which signifies a strong and stable interaction with the target protein. Similarly, 3-(3-chloro-1H-1,2,4-triazol-1-yl)adamantane-1-carboxylic acid displayed a notable binding affinity of -10.85 kcal/mol and an RMSD of 1.60 Å, suggesting a potentially potent inhibitory effect on PPARGC1A.Furthermore, the 7-[(3-Methoxyphenyl)methyl]-2-pyrrolidin-1-yl-3,5,6,8-tetrahydropyrido[3,4-d]pyrimidin-4-one exhibited an exceptionally high binding affinity of -12.34 kcal/mol along with an RMSD of 1.64 Å, making it a prime candidate for further investigation due to its substantial interaction with the protein. Another one, N-(5-fluoro-2-methylphenyl)-2-[4-methyl-6-oxo-2-(pyrrolidin-1-yl)-1,6-dihydropyrimidin-1-yl]acetamide, also showed a significant binding affinity of -11.46 kcal/mol and an RMSD of 1.68 Å, aligning it with strong contenders for PPARGC1A inhibition. Lastly, N-[(2-Fluorophenyl)methyl]-2-(piperidin-1-YL)-5,6,7,8-tetrahydroquinazoline-6-carboxamide recorded a binding affinity of -6.44 kcal/mol and an RMSD of 1.76 Å (**Table 3**). Despite its relatively lower binding affinity compared to the others, its interaction with the protein could be optimized through further modifications to enhance its inhibitory action. These findings collectively underscore the potential of these enhancers, especially the ones with the highest binding affinities, to serve as effective enhancers of PPARGC1A, pending further experimental validation to confirm the computational predictions.

**Table 3:** Molecular docking results for PPARGC1A with screened enhancers.

Enhancers	PubChem IDs	Binding Affinity (kcal/mol)	RMSD
1-(3,4-dihydro-2H-pyrrol-5-yl)-3-(2-methoxyphenyl)-1-(4-methoxyphenyl)urea	7287281	-9.1	1.1
3-(3-chloro-1H-1,2,4-triazol-1-yl)adamantane-1-carboxylic acid	601625	-1-.85	1.60
7-[(3-Methoxyphenyl)methyl]-2-pyrrolidin-1-yl-3,5,6,8-tetrahydropyrido[3,4-d]pyrimidin-4-one	135719671	-12.34	1.64
N-(5-fluoro-2-methylphenyl)-2-[4-methyl-6-oxo-2-(pyrrolidin-1-yl)-1,6-dihydropyrimidin-1-yl]acetamide	49667243	-11.46	1.68
N-[(2-Fluorophenyl)methyl]-2-(piperidin-1-YL)-5,6,7,8-tetrahydroquinazoline-6-carboxamide	71836093	-6.44	1.76

## 4 Discussion

Alzheimer’s disease is a progressive neurodegenerative disease, typically observed in older individuals. While Aβ amyloid plaques and phosphorylated tau proteins have been the focus of most attention, numerous studies indicate that there is a role for oxidative stress in AD pathogenesis (38). However, the precise mechanism by which oxidative stress contributes to AD pathogenesis is still inadequately defined. Intensive investigation of the underlying mechanism of oxidative stress in AD may be crucial for developing novel therapeutic interventions (39). On the other hand, the brain mostly relies on glucose for energy, but in AD glucose metabolism is intensely decreased, probably owing, at least in part, to oxidative damage to enzymes involved in glycolysis, the tricarboxylic acid cycle and ATP biosynthesis (5). This defect likely results in substantial part from oxidative damage to key proteins in glycolysis, the TCA cycle and ATP synthase. However, how do oxidative stress related genes and glucose metabolism genes cross talk to mediate AD? Some studies suggest a role for oxidative stress related genes in AD (39) while some other studies point to specific roles for glucometabolic genes in the onset and progression of AD, on the basis of glucometabolic-associated DEGs and key genes (40). However, in our study we worked on understanding possible synergies of these in mediating AD.

In our research, we identified 21 oxidative stress and glucose metabolism related DEGs in AD patients through bioinformatics analysis. Via the PPI network and plotting ROC curve analysis, we identified 5 oxidative stress and glucose metabolism-related hub genes (AKT1, PPARGC1A, PPARA, PDK1, ACACB) in AD patients with good diagnostic values in the training dataset GSE125583 and external validation dataset GSE173955. These top 5 hub genes are involved in the longevity related pathway, glucagon signaling pathway, insulin resistance pathway and Alzheimer disease pathway. The integrative analysis of OSGMGs, DEGs, and WGCNA datasets, followed by ROC analysis and PPI network evaluation, highlighted the importance of hub genes AKT1 and PPARGC1A. These genes showed high predictive accuracy with AUC values exceeding 0.70, underscoring their potential in AD pathogenesis and progression. The robustness of AKT1 and PPARGC1A as hub genes is further accentuated by their pivotal roles in neuronal survival and energy metabolism, respectively, which are critical processes implicated in AD pathophysiology.

Comparatively, our findings are consistent with existing literature indicating the crucial roles of AKT1 and PPARGC1A in neurodegenerative diseases. Previous studies have implicated AKT1 in neuronal cell survival pathways, and dysregulation of PPARGC1A has been associated with mitochondrial dysfunction in AD. AKT1 influences various cellular processes, including metabolism, growth, proliferation, survival, transcription, and protein synthesis, which are critical in maintaining neuronal health and function (41). In our study, we observed an upregulation of AKT1 expression in AD, aligning with findings from previous research (17). Yang et al. (42) reported that Akt is increasingly activated in the neuronal cells of AD patients. Incessant activation of the PI3K/Akt pathway suppresses mTOR inhibition and the protective effect of FOXO signaling, thus aggravating the impact of Tau hyperphosphorylation and Aβ deposition, cognition impairment, and synaptic damage. Due to the significant effect of the Akt signaling pathway on AD deterioration, understanding the dynamics of AKT1 upregulation could therefore provide critical insights into developing targeted therapies as we suggest with the inhibitor molecules that we identified in this article. Machine learning-based virtual screening helped us identified molecular patterns, docking and potential efficacy of multiple small molecules that could interacts with AKT1. Interestingly, N-Tosylcyclohexanecarboxamide exhibited the highest binding affinity for AKT1, suggesting it as a lead molecule for further development.

Additionally, PPARGC1A also referred to as PGC- 1α, involved in the regulation of cellular mitochondrial biogenesis and energy metabolism, functions which are impaired in AD. Reduction of PPARGC1A, has been shown to modulate neuronal oxidative stress and mitochondrial function besides being downregulated in brains of AD patients (43). As previously mentioned, PPARGC1A is involved in regulating energy homeostasis, in addition to other aspects of the disease process, such as inflammatory gene expression and synaptic function, which are impaired in AD patients (44). For instance, research by Zheng et al. (45) has indicated that PPARGC1A overexpression could prevent amyloid-beta disruption of both mitochondrial capabilities and oxidative stress in neurons and therefore enhancing the activity of PPARGC1A might possibly provide a form of protection against the development of AD. Similarly, interventions that help activate this PPARGC1A have been demonstrated to elicit enhanced cognitive performance and lower neurodegeneration in AD models, thus lending further credence to the role of this pathway as a therapeutic target in AD (46). In this study we also identified high binding affinities molecules targeting PPARGC1A, particularly 7-[(3-Methoxyphenyl)methyl]-2-pyrrolidin-1-yl-3,5,6,8-tetrahydropyrido[3,4-d]pyrimidin-4-one, underscore their potential as effective PPARGC1A enhancer. Despite the promising binding affinities observed through molecular docking, it is important to note that these simulations are limited by assumptions such as rigid protein structures and do not account for the full complexity of biological systems, including solvation, protein dynamics, and cellular environments. Furthermore, the potential for off-target effects or adverse pharmacological interactions of the screened small molecules was not assessed in this study, and future work will require detailed ADMET profiling and experimental validation to fully evaluate their therapeutic potential.

We were able to show that molecular weight and LogP values are significant features for molecule activity and established their role for the AD drug discovery process. The Random Forest model emerged as the most effective, with an AUC of 0.990, suggesting its potential application in high-throughput screening assays. PCA highlighted the dimensionality reduction in molecular feature space, emphasizing the significance of certain features over others. The dominance of the first principal component in explaining the variance suggests that a few molecular descriptors may hold the key to determining the activity of small molecules against AD targets. Although the Random Forest model demonstrated high predictive performance on our curated dataset, we recognize its reliance on predefined molecular descriptors and the inherent limitations this poses in capturing the full complexity of drug behavior in biological systems. Future efforts will focus on external validation and exploring more advanced modeling techniques, including integration of biological features and deep learning approaches, to enhance predictive accuracy and generalizability.

Our research has certain limitations that must be acknowledged. While the bioinformatics and machine learning approaches provide a powerful preliminary screening tool, experimental validation *in vivo* and *in vitro* is imperative to confirm the therapeutic potential of these molecules. Moreover, the complexity of AD pathogenesis suggests that monotherapy targeting a single protein may be insufficient. While AKT1 and PPARGC1A are not brain-exclusive genes, RNA expression data from the Human Protein Atlas (HPA) confirm their widespread expression across key regions of the human brain, including the hippocampus and cortex—areas central to Alzheimer’s disease pathology (47). This supports their biological relevance in the CNS. Notably, AKT1 has been shown to regulate neuronal survival and synaptic plasticity, while PPARGC1A is critical for mitochondrial biogenesis and neuronal energy metabolism—both key processes impaired in AD (41, 44). However, we acknowledge the necessity of cell-type–specific analyses in future studies and propose that subsequent work integrate tissue-aware functional networks such as GIANT and HumanBase, which model context-specific gene interactions to enhance the precision of computational predictions (48, 49). Given that AKT1 and PPARGC1A are broadly expressed and serve as central signaling hubs across multiple tissues, systemic targeting of these genes may lack the specificity required for safe therapeutic application. As such, these findings should be considered primarily as a proof-of-concept, illustrating the potential of our integrative computational pipeline for discovering and prioritizing candidate genes and small molecules in Alzheimer’s disease, rather than as immediate therapeutic leads.

Therefore, future studies could focus on multi-targeted drug approaches and combinatorial therapies that may offer more comprehensive treatment strategies. To sum up, our integrative methodology exemplifies the utility of combining computational and experimental approaches to hasten the discovery of potential therapeutic agents for complex diseases like AD. This work provides direction for follow-up experimental work and highlights the possibility of utilizing machine learning to improve drug discovery. The implications of these findings are optimistic for the use of precision medicine approaches in the management of AD.

## 5 Conclusion

In conclusion, we have identified significant genes that link the pathways of oxidative stress and glucose metabolism in AD based on our comprehensive bioinformatics analysis. We have substantiated the significance of AKT1 and PPARGC1A in maintaining neuronal integrity and metabolism, both of which are disrupted in AD. Understanding AKT1’s participation in the cell survival signaling networks and the influence of PPARGC1A on mitochondria helps elucidate AD at the molecular level. These findings support and extend those previous studies by confirming that modulation of oxidative stress and promotion of metabolism are potential approaches to AD prevention. In our study, machine learning has not only helped enhance the predictive accuracy of biomarker performance but also supported the discovery of new small molecules by means of virtual screening. This approach yielded promising leads, such as N-Tosylcyclohexanecarboxamide and 7-[(3-Methoxyphenyl)methyl]-2-pyrrolidin-1-yl-3,5,6,8-tetrahydropyrido[3,4-d]pyrimidin-4-one, which demonstrated significant binding affinities to AKT1 and PPARGC1A, respectively. Additionally, given the multifactorial nature of AD, a single-target therapeutic strategy may not be sufficient to address the complexities of the disease. Future research should, therefore, consider multi-target approaches and the potential for combination therapies to provide a more effective treatment paradigm for AD. Our work lays a solid foundation for the further exploration of oxidative stress and glucose metabolism in AD and opens avenues for the development of targeted therapeutics. By harnessing the power of computational biology and machine learning, we move closer to expanding the therapeutic options for treating Alzheimer’s disease.

## Data Availability

The original contributions presented in the study are included in the article/**Supplementary Material**. Further inquiries can be directed to the corresponding author.
