# Supplementary material for: Integrative bioinformatics and machine learning approaches reveal oxidative stress and glucose metabolism related genes as therapeutic targets and drug candidates in Alzheimer’s disease
Source: Front Immunol. 2025 Jun 26;16:1572468. doi: 10.3389/fimmu.2025.1572468 (PMC12241127; doi:10.3389/fimmu.2025.1572468)
Supplement: Supplementary file 1 [file DataSheet1.docx]

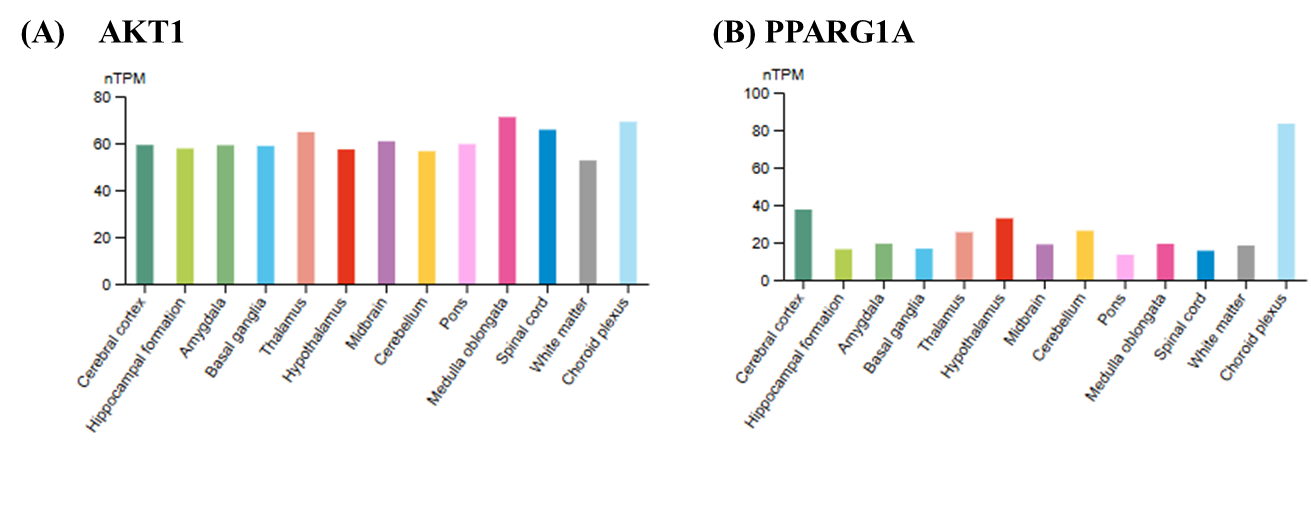


**Supplementary Figure S1.** RNA expression profiles of AKT1 and PPARGC1A in the human brain based on data from the Human Protein Atlas (<https://www.proteinatlas.org/>). Both genes are detectably expressed in all major anatomical regions of the brain. Expression of AKT1 is broadly consistent across regions, while PPARGC1A shows high expression particularly in the choroid plexus, suggesting CNS relevance. This supports their potential involvement in Alzheimer’s disease

**Supplementary Figure S2.** RNA expression profiles of AKT1 and PPARGC1A in the human brain based on data from PMID: **26687838**. Both genes are detectably expressed in all major cell types of the brain.
